# Unicornuate uterus with a rudimentary non-communicating cavitary horn in association with VACTERL association: case report

**DOI:** 10.1186/s12905-019-0768-4

**Published:** 2019-05-30

**Authors:** Rawan A. Obeidat, Abdelwahab J. Aleshawi, Nour A. Tashtush, Haya Alsarawi

**Affiliations:** 1 Department of Obstetrics & Gynecology, Faculty of Medicine, Jordan University of Science & Technology, Irbid, Jordan; 2 Department of General Surgery, Faculty of Medicine, Jordan University of Science & Technology, Irbid, Jordan; 3 Department Pediatrics and Neonatology, Faculty of Medicine, Jordan University of Science & Technology, Irbid, 22110 Jordan

**Keywords:** Imperforate anus, Müllerian duct, Secondary dysmenorrhea, Unicornuate uterus, VACTERL association

## Abstract

**Background:**

The unicornuate uterus is caused by abnormal or failed development of one Müllerian duct. Unicornuate uteri with functioning non-communicating rudimentary horns are susceptible to many gynaecologic and obstetric complications such as hematometra, endometriosis and ectopic pregnancy and thus surgical resection is usually recommended..

**Case presentation:**

We report a rare case of a unicornuate right uterus with rudimentary non-communicating (functional) cavitary left horn (class U4a) in a 17-year-old girl who was diagnosed with VACTERL association. She was presented to our centre with 3 years history of secondary sever dysmenorrhea. Pelvic magnetic resonance imaging revealed a normal uterus on the right side, a 7 × 8 cm left endometrioma, a tortuous dilated fluid-filled structure in the left hemipelvis, mostly represented left-sided hematosalpinx, and a well-defined lesion with thick enhancing wall in the left hemipelvis measuring 6.7 × 5.7 × 5.6 cm with a similar enhancement to the uterus in the right. She underwent laparotomy that showed a right unicornuate uterus with a normal cervix and a rudimentary non-communicating distended left horn. In addition, there was a left endometrioma and left hematosalpinx. Resection of the left communicating horn, left salpingectomy and left ovarian cystectomy were performed. The right tube and both ovaries were preserved. At 9-months follow up, the patient had a regular period and the pain subsided completely.

**Conclusion:**

We report yet the second case of VACTERL association and unicornuate uterus with non-communicating functional rudimentary horn, in hope of expanding the knowledge of a rare occurrence. This case also highlights the importance of considering the diagnosis of Müllerian duct anomalies in patients with a history of other anomalies, and/or history of early-age secondary dysmenorrhea.

## Background

The true incidence of Müllerian duct anomalies (MDAs) are believed to be between 0.1 and 3.8% [1]. Although its incidence may be as high as 25% in women with recurrent miscarriages and subfertility [2]. MDAs are congenital anatomic abnormalities of the female genital tract that arise from nondevelopment or nonfusion of the Müllerian ducts or failed resorption of the uterine septum [3, 4]. MDAs may lead to symptoms such as pelvic pain, dysmenorrhea, abnormal bleeding at the time of menarche, recurrent pregnancy loss, and/or premature delivery [4]. Patients with MDAs are at increased risk of having renal, skeletal, or abdominal wall abnormalities [5]; these abnormalities should also be identified and documented.

The European Society of Human Reproduction and Embryology (ESHRE) and the European Society for Gynaecological Endoscopy (ESGE) classification system for female genital malformations organizes uterine anomalies into six main classes: Class U0 involves all cases with a normal uterus. Class U1 or dysmorphic uterus incorporates all cases with a normal uterine outline but with an abnormal shape endometrial cavity excluding septa. Septate uterus is the class U2 anomaly. Class U3 or bicorporeal uterus comprises all cases of fusion defects. Class U4 or hemi-uterus includes all cases of the unilateral formed uterus. Class U5 or aplastic uterus incorporates all cases of uterine aplasia. Class U6 is kept for still unclassified cases. Cervical and vaginal anomalies are classified in independent supplementary sub-classes [6].

We report a rare case of a unicornuate right uterus with rudimentary non-communicating (functional) cavitary left horn (class U4a) in link with (Vertebral defects, Anal atresia, Cardiac defects, Tracheo-Esophageal fistula, Renal anomalies, and Limb abnormalities) VACTERL association.

## Case presentation

A 17-year-old girl; a known case of a repaired high imperforate anus, repaired type-C tracheoesophageal malformation, and left renal agenesis; presented to King Abdullah University Hospital (KAUH) complaining of dysmenorrhea for 3 years duration. She was 14-year-old when she had her menarche. The menstrual cycle was irregular and associated with severe dysmenorrhea. She had the dysmenorrhea 4 days before the period, during the period and lasted 1 week after. The pain was slightly relieved by analgesics. The complaint was associated with vomiting, anorexia, and general fatigability. Prior medical and surgical history included two-steps primary anoplasty repair for imperforate anus which involved a temporary colostomy creation followed by posterior sagittal anorectoplasty. The tracheoesophageal fistula was repaired by resection of the fistula and anastomosis of the esophageal limbs. Examination revealed abdominal mass and tenderness.

Laboratory investigations were conducted and revealed elevated levels of cancer antigen 125 (CA 125) (241 U/ml) and CA 19–9 (67 U/ml). Other tests including complete blood count, kidney function test, CA 15.3, alpha-fetoprotein, lactate dehydrogenase, and human chorionic gonadotropin (hCG) were all within normal levels. Also, urinalysis and culture disclosed no abnormalities.

**Fig. 1:**
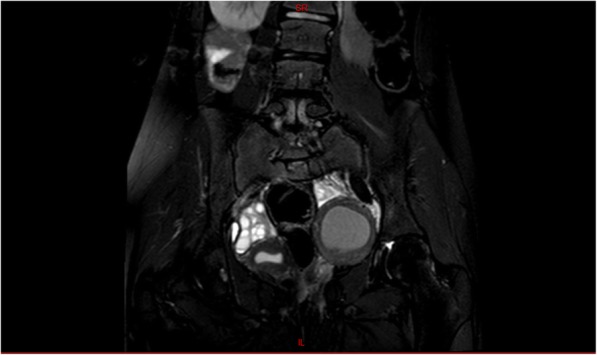
Pelvic magnetic resonance imaging: T2 signal magnetic resonance imaging indicated the presence of left hematometra with small right uterus. Both ovaries were demonstrated

**Fig. 2:**
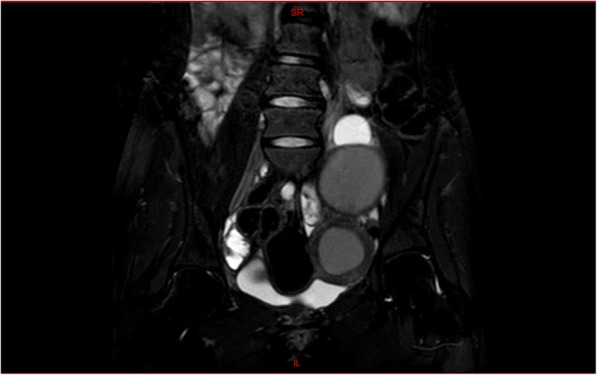
Pelvic magnetic resonance imaging: T2 signal magnetic resonance imaging indicated the presence of left endometrioma above the hematometra

Abdominal and pelvic ultrasound was performed and showed a thick-wall left pelvic mass measuring 6 × 6 cm and another 7 × 8 cm left pelvic mass mostly endometrioma. Renal ultrasound confirmed the solitary right kidney with compensatory hypertrophy. Pelvic magnetic resonance imaging (MRI) revealed a normal uterus on the right side, a normal right ovary, a 7 × 8 cm left endometrioma, a tortuous dilated fluid-filled structure in the left hemipelvis mostly represented left-sided hematosalpinx, and a well-defined lesion with thick enhancing wall in the left hemipelvis measuring 6.7 × 5.7 × 5.6 cm with similar enhancement to the uterus in the right suggestive of MDA probably double uterus obstructed on the left side with large hematometra. (Figs. 1 and 2).

**Fig. 3:**
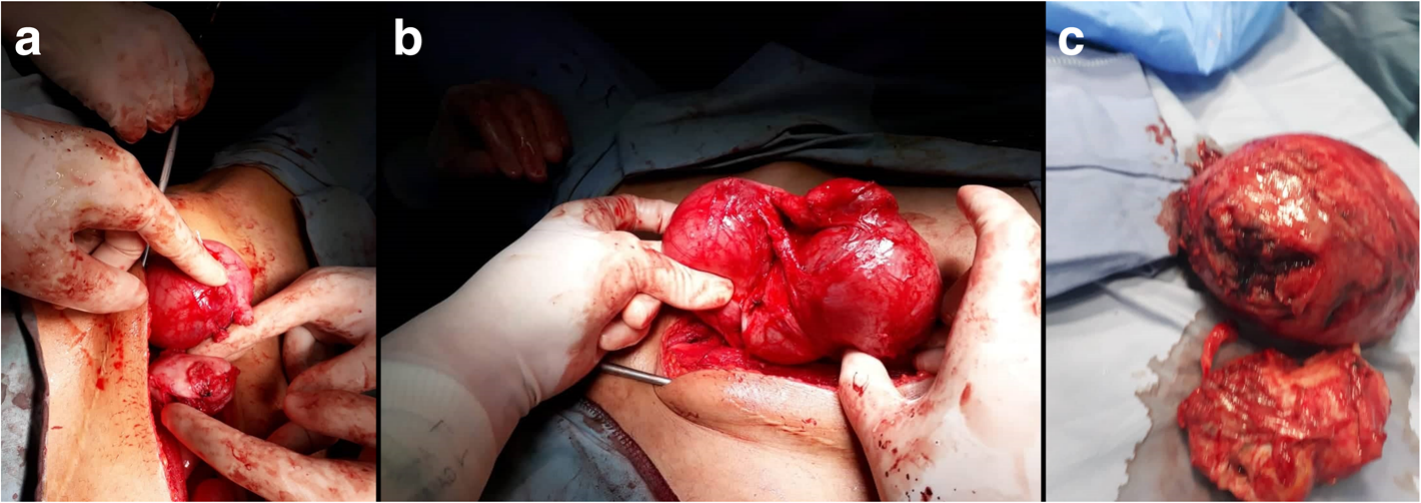
**a**, **b**, **c** Intraoperative findings of large congested left rudimentary non-communicating cavitary horn with right unicornuate uterus

Initially the patient had diagnostic laparoscopy using the open technique, however, the patient had extensive adhesions and thus laparotomy with adhesiolysis was performed. At laparotomy, there was a right unicornuate uterus with a normal cervix and a non-communicating distended left rudimentary horn. Additionally, there was a left hematosalpinx of the left tube which was connected to the left horn, and a left endometrioma. The right ovary and tube looked normal. The single right ureter was also identified. The left non-communicating horn was resected. In addition, left salpingectomy and left ovarian cystectomy were performed. The right uterus and both ovaries were preserved. The diagnosis of class U4a uterine anomaly (unicornuate right uterus with rudimentary non-communicating cavitary left horn) was established (Fig. 3). No intraoperative or immediate postoperative complications were detected. The patient was discharged on the fourth postoperative day. Histopathological examination of the specimen was reported as uterine rudimentary horn with unremarkable myometrium and secretory endometrium, and left endometriotic cyst. At 9 months follow up, the patient had regular menstrual cycles and the pain subsided completely.

## Discussion and conclusion

In embryological development, the Müllerian (or paramesonephric) ducts are first identifiable at 5–6 weeks gestation when they begin to grow mediocaudally toward the urogenital sinus. The cranial vertical part and the mid horizontal part of each duct develop into fimbria and fallopian tube while the caudal part of the duct conjoins in the midlines with its contralateral side to create uterovaginal canal that will develop to the uterus, cervix, and upper vagina. Initially, the two Müllerian ducts are composed of solid tissue, and then internal canalization of each duct produces two canals separated by a septum that is usually regressed at around 20 weeks. The uterine endometrium is derived from the lining of the fused Müllerian ducts, whereas the endometrial stroma and myometrium are derived from adjacent mesenchyme [7–9].

Embryologically, the urinary system and the genital system are closely linked. Both develop from the intermediate mesoderm along the posterior wall of the abdominal cavity, and initially, the excretory ducts of both systems enter a common cavity, the cloaca. Three slightly overlapping kidney systems are formed in a cranial-to-caudal sequence during intrauterine life in humans: the pronephros and mesonephros which are transitory structures but critical to the development of the metanephros. The paired mesonephric ducts drain the mesonephros into the cloaca and are necessary for lower vaginal formation. The permanent kidneys develop from two sources; the ureteric bud and the metanephrogenic blastema. The ureteric bud is a diverticulum from the mesonephric duct near its entrance into the cloaca and it is the primordium of the ureter, renal pelvis, calices, and collecting tubules [7, 9].

During the fourth to seventh weeks of development, the cloaca divides into the urogenital sinus anteriorly and the anal canal posteriorly. The urogenital sinus gives rise to lower vagina, bladder (except trigon) and urethra. The trigone is derived from the caudal ends of the mesonephric ducts. The ovaries develop from primitive germ cells, mesothelium of the posterior abdominal wall and adjacent mesenchyme and are independent of Müllerian duct differentiation. Therefore, the ovaries are usually normal in a patient with MDAs [7–9].

The unicornuate uterus (Class U4) is a result of abnormal or failed development of one Müllerian duct. It accounts for approximately 2.4–13% of all Müllerian anomalies [10] and is divided into two sub-classes depending on the presence or not of a functional rudimentary cavity. Class U4a or hemi-uterus with a rudimentary (functional) cavity characterized by the presence of a communicating or non-communicating functional contralateral horn. Class U4b or hemi-uterus without rudimentary (functional) cavity characterized either by the presence of non-functional contralateral uterine horn or by aplasia of the contralateral part [6].

Clinically, non-communicating rudimentary horns with functional endometrium are the most significant subtype. The correct diagnosis of this entity has important clinical implications as they are likely to be associated with dysmenorrhea and pelvic pain from haematometra or from endometriosis due to retrograde menstruation [11]. Furthermore, pregnancies in these rudimentary horns can occur following transperitoneal migration of sperm or zygote and generally will result in a life-threatening uterine rupture [12]. Whenever diagnosed, surgical excision (preferably laparoscopic) of the functional rudimentary horn is recommended even if the horn is communicating.

The acronym *VATER* association was first described by Quan which followed by the acronym VACTERL described by Baumann [13, 14]. This association comprises a group of birth defects which tend to co-occur. These defects are vertebral anomalies, anorectal malformations, cardiovascular anomalies, tracheoesophageal fistula, esophageal atresia, renal anomalies, and limb defects [13, 14]. The presence of at least three of the aforementioned anomalies is diagnostic for VACTERL association [14]. In the presenting case, the patient was born with imperforate anus, tracheoesophageal fistula, esophageal atresia, and left renal agenesis.

To our knowledge, this is the second documented case describes a VACTERL association with the coexistence of unicornuate uterus with a non-communicating rudimentary functional horn. Nunes et al. reported the first case in a 28-year-old nullipara female known to have a missing vertebra, imperforate anus, anovaginal fistula, right renal agenesis, and an extra digit on one hand [5]. She had a regular cycle since menarche at age 15. She presented with a 6-month history of progressive constipation and sciatica followed by acute urinary retention which was revealed to be due to hematometra/hematosalpinx of a non-communicating right uterine horn and fallopian tube [5]. Jessel et al. described a case of distal vaginal agenesis and right unicornuate uterus with left non-obstructed rudimentary horn in a 14-year-old girl with a known history of VACTERL association and repaired imperforate anus [15].

Heinonen reported no VACTERL association in his series with 42 cases of unicornuate uteri [16]. Heinonen reported the presence of Kidney abnormalities, bony anomalies, auditory defect, Hirschsprung’s disease, an absence of the gallbladder, and annular pancreas [16].

To finish, the development of the urinary system is closely related to the genital tract and anomalies of these organs are often coexisting. Less commonly, MDAs may coexist with developmental anomalies of the distal gastrointestinal tract particularly cloaca anomaly [17]. A retrospective study of female patients treated for imperforate anus revealed that primary vaginal anomalies occurred in 22 of 72 (32%) patients assessed, and uterine anomalies occurred in 18 of 51 (35%) patients assessed. A bicornuate uterus and uterus didelphys were the most common abnormalities [18]. In our case, and the case described by Nunes et al., the patients had a unicornuate uterus with rudimentary horn and the diagnosis of MDAs was not recognized until they develop symptoms later. Hence, it is important that an experienced gynaecologist is involved at an early stage in the care of female infants with anorectal malformations.

We report yet the second case of VACTERL association and a unicornuate uterus with non-communicating functional rudimentary horn, in hope of expanding the knowledge of a rare occurrence. This case also highlights the importance of considering the diagnosis of MDAs in patients with a history of other anomalies, and/or history of early-age secondary dysmenorrhea.

## Abbreviations

CA: cancer antigen
MDAs: Müllerian duct anomalies
VACTERL: Vertebral defects, Anal atresia, Cardiac defects, Tracheo-Esophageal fistula, Renal anomalies, and Limb abnormalities
